# Diabetes-dependent quality of life (ADDQOL) and affecting factors in patients with diabetes mellitus type 2 in Greece

**DOI:** 10.1186/s13104-015-1782-8

**Published:** 2015-12-15

**Authors:** Athanasia K. Papazafiropoulou, Florentia Bakomitrou, Aikaterini Trikallinou, Asimina Ganotopoulou, Chris Verras, George Christofilidis, Stavros Bousboulas, Αndreas Μelidonis

**Affiliations:** 1st Department of Internal Medicine and Diabetes Center, Tzaneio General Hospital of Piraeus, 1 Zanni and Afentouli Street, 185 36 Piraeus, Greece; 3rd Internal Medicine Department and Diabetes Center, General Hospital of Nikaia, Athens, Greece

**Keywords:** Type 2 diabetes, Quality of life, Health status, Dietary habits, Age

## Abstract

**Background:**

Diabetes mellitus type 2 (T2D) is a chronic metabolic disease with a great impact on health status and quality of life (QoL) in terms of physical, social, and psychological well-being. The aim of the present study was to measure diabetes-dependent QoL and affecting factors in patients with T2D.

**Methods:**

Study population was consisted by 258 subjects with T2D attending diabetic outpatient clinics of General Hospitals of Piraeus “Tzaneio” and Nikaia “Ag.Panteleimon” during September–December 2014. The Audit of Diabetes-Dependent Quality of Life questionnaire was carried out in all study participants.

**Results:**

Diabetes mellitus type 2 had a negative impact to QoL in 37.3 % of the study participants while 32.9 % believed that their life would have been better without the presence of T2D. Diabetes had negative impact on working life (−1.3 ± 0.6), health status (−1.3 ± 0.2), family (−1.3 ± 0.6) and sexual life (−1.3 ± 0.3), future perspectives (−1.3 ± 0.4) and dietary habits (−1.7 ± 0.2). The results of logistic regression analysis showed that QoL was related with age [odds ratio (OR) 0.94, 95 % confidence intervals (CIs) 0.91–1.98, P = 0.008] and marital status (OR 0.43, 95 %CIs 0.21–0.90, P = 0.03).

**Conclusions:**

The results of the present study showed that T2D per se has a negative impact to patient’s QoL most of all affecting working life, health status, family and sexual life, future perspectives and dietary habits. Age and marital status were the only determinants of QoL.

## Background

Type 2 diabetes mellitus (T2D) is a chronic metabolic disorder with a currently estimated global prevalence of 8.3 % [1]. While it’s incidence has increased rapidly during the past few decades worldwide, from approximately 35 million people affected in 1985 to about 171 million in 2000 [2]. T2D affects both health and quality of life (QoL). There are a number of studies showing that QoL is reduced in T2D patients compared to the general population [3] and also QoL is lower than in patients with other chronic disease entities [4]. A multinational study showed that diabetes has a negative impact on general health, and poor QoL is associated with adverse outcomes, including increased mortality in T2D patients [4, 5]. Also, presence of diabetic complications has been reported to have a significant influence on the QoL [6, 7]. On the other hand, strict glycemic control that is required to prevent diabetic complications seems to have an important impact on QoL in T2D patients [8]. Therefore, it is important to identify factors that affect diabetes-related QoL in order to manage them properly and improve QoL in diabetic patients [9, 10].

Various instruments assessing the QoL related to diabetes have been used. Among diabetes-specific QoL measures, the Audit of Diabetes Dependent Quality of Life (ADDQOL) is a widely-used instrument of diabetes-specific QoL that assesses an individual’s perceptions of the impact of diabetes on their QoL [11]. ADDQOL is a well validated scale and, in previous studies, has showed an important negative impact of diabetes on all domains of a patient’s life [11, 12]. However, to the best of our knowledge there is no data available evaluating patients’ diabetes-related QoL from our country. Therefore, the purpose of this study was to assess QoL as well as factors associated with the diabetes related QoL measured by the ADDQOL in Greek patients with T2D.

## Methods

### Setting and participants

A cross-sectional study was conducted between September and December 2014 using the ADDQOL questionnaire. A total of 258 participants with T2D attending the two diabetic outpatient clinics of General Hospitals of Piraeus “Tzaneio” and Nikaia “Ag.Panteleimon” were enrolled into the study. Basic demographic information, sociodemographic characteristics, medical history and duration of diabetes, glycemic control and existing diabetic complications were obtained at the time of the visit. Exclusion criteria were history or current treatment for mental disorder.

The ethical committee of General Hospitals of Piraeus “Tzaneio” and Nikaia “Ag.Panteleimon” provided approval for this study. All participants gave their written consent before enrollment into the study. Where assistance was needed in completing the questionnaire, this was given by physicians, who were trained in the use of the ADDQOL questionnaire. The study was in accordance with the Helsinki declaration.

### Instrument

The ADDQOL consists of two overview items; one measures generic overall QoL and a further 19 items are concerned with the impact of diabetes on specific aspects of life. The 19 life domains are as follows: leisure activities, working life, local or long-distance journeys, holidays, physical health, family life, friendships and social life, close personal relationships, sex life, physical appearance, self-confidence, motivation to achieve things, people’s reactions, feelings about the future, financial situation, living conditions, dependence on others, freedom to eat, and freedom to drink. These 19 domains ask the respondents to evaluate how their life would be if they did not have diabetes. The scales range from −3 to +1 for 19 life domains (impact rating) and from 0 to +3 in attributed importance (importance rating). A weighted score for each domain is calculated as a multiplier of impact rating and importance rating (ranging from −9 to +3). Lower scores reflect poorer QoL. Finally, a mean weighted impact score (ADDQOL score) is calculated for the entire scale across all applicable domains [11, 12].

### Statistical analysis

Statistical analysis was performed using programs available in the SPSS statistical package (SPSS 19.0, Chicago, USA). Data are shown as mean ± standard deviation (SD), unless it is stated otherwise. A two sample t test was used to assess differences in continuous variables, while a Chi-square test was used for categorical variables. Logistic regression analysis was used to assess the influence of sociodemographic and diabetic characteristics of study patients on their QoL by using the ADDQOL. Patients were divided into two groups by the ADDQOL score by using quartiles; the first group in the lower quartile was considered as having lower QoL. Such a cutoff strategy was previously applied in the literature [13, 14]. P < 0.05 (two-tailed) was considered statistically significant.

## Results

Demographic and clinical characteristics of study population are presented in Table 1. About 52 % of the study population were men (n = 131), mean aged 58.1 ± 11.1 years. The majority of the respondents were married (54.3 %), had secondary education (62.4 %) and monthly income ≤700 € (50.7 %).

**Table 1:** Demographic and clinical characteristics of the study population

Variables	N (%)
Gender (males)	131 (51.8)
Age ± SD (years)	58.1 ± 11.1
Diabetes duration ± SD (years)	10.3 ± 3.8
HbA1c ± SD (%)	7.1 ± 1.4
Body mass index ± SD (kg/m^2^)	31.5 ± 7.1
Monthly income (Euros)	
≤700	130 (50.7)
700–1000	52 (20.3)
≥3000	76 (29.0)
Educational level	
Low	75 (29.3)
High	183 (70.7)
Marital status	
Never married	36 (14.3)
Married	211 (82.7)
Divorced	11 (4.0)
Smoking status	
Non or ex smoker	162 (63.4)
Current smoker	96 (36.6)
Oral antidiabetic therapy (yes)	199 (77.5)
Insulin therapy (yes)	115 (44.8)
Coronary artery disease (yes)	36 (14.2)
Cerebrovascular disease (yes)	10 (4.0)
Peripheral arterial disease (yes)	11 (4.5)
Retinopathy (yes)	25 (9.5)
Neuropathy (yes)	13 (4.9)
Chronic kidney disease (yes)	12 (4.6)

Duration of diabetes was 10.3 ± 3.8 years, HbA1c 7.1 ± 1.4 % and body-mass index (BMI) 31.5 ± 7.1 kg/m^2^. Of the study participants, 77.5 % were on oral antidiabetic treatment while 44.8 % on insulin therapy. Regarding diabetic complications; 14.2 % had coronary artery disease, 4.0 % cerebrovascular disease, 4.5 % peripheral arterial disease, 9.5 % retinopathy, 4.9 % neuropathy and 4.6 % nephropathy (Table 1).

Diabetes mellitus type 2 had a negative impact to QoL in 37.3 % of the study participants while 32.9 % believed that their life would have been better without the presence of T2D. The ADDQOL score was calculated in a range of −9.0 to 0 on a defined range from −9 to +3. The median ADDQOL score was calculated at −2.7. Then lower quartile cutoff was calculated at −3.0, 149 (57.7 %) patients with T2D reported an ADDQOL score of −3.0 or more, and 109 (42.3 %) patients had an ADDQOL score of less than −3.0 (lower QoL). It is noteworthy that five patients (1.9 %) reported an ADDQOL score of 0, which means that their QoL was not affected by diabetes at all.

The distribution of responses and the weights assigned to the impact ratings are shown in Table 2. Diabetes had the greatest impact on “freedom to eat” (mean impact rating: −1.7 ± 1.0) and the least impact on “physical appearance” (mean −1.0 ± 1.1), “motivation” (mean −1.0 ± 1.1), “people’s reaction” (mean −1.0 ± 1.0), “financial situation” (mean −1.0 ± 1.3) and “dependence on others” (mean −1.0 ± 1.0). “Family life” was rated as the most important (mean 2.6 ± 0.8) while “freedom to drink” was rated as the least important (mean 1.5 ± 1.1) QoL domains, respectively, for the study participants. After considering weighting, “freedom to eat” (mean −4.2 ± 3.2) was the most and “people’s reaction” (mean −1.6 ± 2.4) was the least affected QoL domains, respectively (Table 2).

**Table 2:** Distribution of response by impact and importance rating together with weighted impact score

Domain	Impact rating	Mean ± SD importance rating	Weighted impact score
Leisure activities	−1.1 ± 1.0	1.9 ± 0.8	−2.3 ± 2.6
Working life	−1.3 ± 1.0	2.2 ± 0.9	−2.9 ± 2.9
Journeys	−1.1 ± 0.2	1.8 ± 0.9	−2.4 ± 2.7
Holidays	−1.2 ± 1.1	1.8 ± 0.9	−2.4 ± 2.7
Physical health	−1.3 ± 1.0	2.0 ± 0.8	−2.8 ± 2.9
Family life	−1.3 ± 1.0	2.6 ± 0.8	−3.2 ± 3.1
Friendship and social life	−1.1 ± 1.1	2.3 ± 0.8	−2.4 ± 2.9
Personal relationship	−1.2 ± 1.2	2.2 ± 0.9	−2.6 ± 3.2
Sex life	−1.3 ± 1.1	2.1 ± 0.9	−2.8 ± 3.2
Physical appearance	−1.0 ± 1.1	1.9 ± 0.8	−2.0 ± 2.8
Self-confidence	−1.2 ± 1.1	2.3 ± 0.8	−2.9 ± 3.1
Motivation	−1.0 ± 1.1	2.3 ± 0.8	−2.7 ± 3.1
People’s reaction	−1.0 ± 1.0	2.1 ± 0.8	−1.6 ± 2.4
Feelings about future	−1.3 ± 1.3	2.3 ± 0.7	−3.2 ± 4.1
Financial situation	−1.0 ± 1.3	2.2 ± 0.8	−2.2 ± 3.8
Living conditions	−1.3 ± 1.1	2.3 ± 0.7	−3.0 ± 3.1
Dependence on others	−1.0 ± 1.0	2.4 ± 0.8	−2.5 ± 3.0
Freedom to eat	−1.7 ± 1.0	2.1 ± 0.9	−4.2 ± 3.2
Freedom to drink	−1.3 ± 1.9	1.5 ± 1.1	−2.5 ± 2.9

Impact rating (conditions without diabetes): −3, very much better; −2, much better; −1, a little better; 0, the same; +1, worse

Importance rating: 0, not at all important; 1, somewhat important; 2, important; 3, very important

Weighted impact score ¼ impact rating (−3 to +1) × importance rating (0–3) ¼ −9 (maximum negative impact of diabetes) to +3 (maximum positive impact of diabetes)

The results of the logistic regression analysis are showed in Table 3. According to the results of the analysis, QoL was related only with age [odds ratio (OR) 0.94, 95 % confidence intervals (CIs) 0.91–0.98, P = 0.008] and marital status (OR 0.43, 95 % CIs 0.21–0.90, P = 0.03). No statistical significant relations were observed between QoL and sex, duration of diabetes, BMI, HbA1c, smoking habits, education level, antidiabetic treatment and diabetic complications.

**Table 3:** Logistic regression analysis: predictors of lower QOL according to the ADDQOL score

Variables	Odds ratio	95 % confidence intervals	P value
Gender (males)	1.09	0.49–2.46	0.82
Age (years)	*0.94*	*0.91–0.98*	*0.008*
Diabetes duration (years)	0.96	0.92–0.98	0.24
HbA1c (%)	0.91	0.74–1.31	0.91
Body mass index (kg/m^2^)	0.98	0.93–1.04	0.49
Monthly income (Euros)	2.61	0.86–7.89	0.09
Educational level	1.42	0.65–3.14	0.38
Marital status	*0.43*	*0.21–0.90*	*0.03*
Smoking status	0.53	0.22–1.24	0.14
Oral antidiabetic therapy (yes)	0.86	0.28–2.65	0.81
Insulin therapy (yes)	0.26	0.22–1.49	0.26
Coronary artery disease (yes)	0.73	0.24–2.24	0.58
Cerebrovascular disease (yes)	0.08	0.22–1.98	0.48
Peripheral arterial disease (yes)	0.32	0.45–1.81	0.19
Retinopathy (yes)	0.14	0.01–1.36	0.09
Neuropathy (yes)	0.26	0.02–2.26	0.21
Chronic kidney disease (yes)	0.12	0.12–1.23	0.10

## Discussion

According to the results of the present study a significant proportion of diabetics in Greece believe that T2D has a negative impact to their QoL. To the best of our knowledge, this is the first study, conducted in our country, in order to assess the impact of T2D on patients’ QoL.

During the recent years QoL has been placed to the center of the management of diabetic patients. Management of T2D patients, except for achieving glycemic control and preventing diabetic complications, gives great importance to diabetic patient QoL since it has a major impact to therapeutic targets [15]. In accordance, the recent guidelines from the American Diabetes Association emphasize the need for a “patient centered” approach of the management of T2D patients in terms of QoL, prevention of diabetic complications and achievement of glycemic targets [15].

In accordance with our results, various studies, in different countries, have reported a negative impact of T2D on QoL [16–19]. QoL in T2D is somewhat lower than in patients with other chronic disease entities [4]. The largest negative impact of T2D observed in the present study was on “freedom to eat”, which is in line with previous studies [11, 12]. Fear of weight gain, high blood glucose levels as well as fear of hypoglycemia affects patient’s dietary behavior [11, 12]. As it has been showed in a recent multicenter study, there is a relationship between diabetes-specific QoL and dietary behavior [19]. Similar results were found in another study where diabetes had the largest impact on “enjoyment of food” and the least impact on “others fussing” [20]. The observation that QoL is impaired in patients with diabetes, especially for the ‘freedom to eat’ domain, indicates that an intervention to improve dietary freedom might be a good way of improving QoL in diabetics [21].

In the present study we observed that lower QoL was related to older age and living alone. In accordance with our results, various studies have showed that QoL is better among people who are at younger age than the eldest ones [3, 11, 12]. However, two recent studies showed that younger age was associated with lower ADDQOL scores in Korean T2D patients [18] and that being younger was associated with a greater negative impact of diabetes on QoL [21]. A possible explanation for this discrepancy might be that diabetics at younger age are afraid in a larger degree for their future and the impact of T2d in their life than the eldest ones. Finally, as it has been showed by previous studies, living alone was significantly correlated with lower QoL. It is well known that QoL is better among married people [3].

It is noteworthy that the results of the present study showed no connection between QoL and diabetic complications. This finding can be, in part, explained by the low prevalence of diabetic complications that we observed in the present study. Several studies have showed that absence of complications was significantly associated with a better QoL among diabetics [22–25]. Furthermore, in another study, a greater negative impact of diabetes on QoL was associated with diabetes complications [21]. Wexler et al. found that patients with symptomatic co-morbidities such as microvascular complications had a substantially reduced QoL, while those without symptoms showed no reduction of their QoL [23]. Another two studies, in different countries, showed that insulin use and diabetes-related complications were significantly associated with poorer QoL [24, 25].

Despite the results of a number of previous studies [11, 12, 18], no association between antidiabetic therapy, especially insulin therapy, as well as duration of diabetes with QoL was found. At this point it must be mentioned that study population had good glycemic control that might affect the impact of different parameters on QoL. However, results similar to ours have also been obtained in the literature [22, 26]. Finally, we found no association between glycemic control (defined as HbA1c) and QoL, a findings that is in agreement with a number of other analyses [4, 27, 28], and in contrast to data reported by Testa et al., who found that improved glycemic control was associated with substantial improvements in QoL [10].

## Conclusion

In conclusion, our results show that T2D per se has a negative impact to patient’s QoL most of all affecting working life, health status, family and sexual life, future perspectives and dietary habits. Age and marital status were the only determinants of QoL in the present study. These findings suggest that we may need different approaches focusing on QoL in the management of T2D patients. However, since our study is cross-sectional, further prospective studies are needed to confirm the results of our study.

## Abbreviations

T2D: diabetes mellitus type 2
QoL: quality of life
ADDQOL: Audit of Diabetes-Dependent Quality of Life
SD: standard deviation
BMI: body-mass index
OR: odds ratio
Cis: confidence intervals
